# Salivary Flow, Tongue-Coating Burden, and Morning Breath Odor: A Cross-Sectional Study

**DOI:** 10.3390/jcm14176072

**Published:** 2025-08-27

**Authors:** Malina Popa, Stefania Dinu, Magda Mihaela Luca, Bogdan Andrei Bumbu, Serban Talpos

**Affiliations:** 1 Department of Pediatric Dentistry, Faculty of Dental Medicine, “Victor Babes” University of Medicine and Pharmacy Timisoara, Eftimie Murgu Square 2, 300041 Timisoara, Romania; popa.malina@umft.ro (M.P.); dinu.stefania@umft.ro (S.D.); 2 Department of Dental Medicine, Faculty of Medicine and Pharmacy, University of Oradea, 410073 Oradea, Romania; 3 Discipline of Oral and Maxillo-Facial Surgery, Faculty of Dental Medicine, “Victor Babes” University of Medicine and Pharmacy Timisoara, 300041 Timisoara, Romania; talpos.serban@umft.ro

**Keywords:** halitosis, saliva, tongue, cross-sectional studies, volatile sulfur compounds

## Abstract

**Background and Objectives:** Morning halitosis undermines social well-being, yet the combined influence of basal salivary flow and tongue coating in healthy adults is unclear. **Methods:** In a cross-sectional study of 92 university students (18–35 years), we measured unstimulated salivary flow rate (uSFR), tongue-coating index (TCI), total volatile sulfur compounds (VSCs; Halimeter^®^), organoleptic score (0–5), and self-perceived halitosis (yes/no) under standardized early-morning conditions. **Results:** Thirty-seven participants (40.2%) reported morning halitosis and showed lower uSFR (0.2 ± 0.1 vs. 0.3 ± 0.1 mL·min^−1^) and higher TCI (2.3 ± 0.5 vs. 1.9 ± 0.4), with higher organoleptic scores (3.4 ± 0.6 vs. 2.1 ± 0.7) and VSCs (272.9 ± 39.8 vs. 163.7 ± 45.9 ppb; all *p* < 0.001). VSCs correlated inversely with uSFR (ρ = −0.58) and positively with TCI (ρ = 0.44). In multivariable models, uSFR (β = −0.53) and TCI (β = 0.31) explained 54% of VSC variance; each 0.1 mL·min^−1^ fall in uSFR increased the odds of self-perceived halitosis 1.9-fold (*p* = 0.001). **Conclusions:** Even among healthy young adults, lower basal saliva and heavier tongue coating are independent contributors to morning malodor. Hydration, daily tongue cleaning, and addressing mouth breathing are pragmatic, first-line strategies.

## 1. Introduction

Halitosis affects an estimated 22–50% of the population, with the prevalence varying by age, geography, and assessment method [1]. Beyond the sensory stigma, individuals with bad breath report clinically meaningful decrements in oral-health-related quality of life [2]. Clinical management has shifted from masking odor to modifying its ecological drivers. Microbiome-targeted approaches show promise, although evidence remains short-term and low-certainty [3].

Day-to-day control still relies on chemotherapeutic rinses and mechanical tongue cleaning, which reduce organoleptic scores and VSCs in controlled trials [4,5,6,7]. Etiologically, the dorsum of the tongue and inflamed periodontal niches are central: patients with gingivitis/periodontitis often show two- to three-fold-higher VSCs than healthy controls [8,9,10,11]. At the same time, saliva provides natural defense by diluting substrates, buffering pH, and delivering antimicrobial factors; reduced unstimulated flow is linked to a higher VSC burden [12,13].

Saliva provides the principal physiological antidote: flow dilutes sulfur precursors, buffers pH, and delivers antimicrobial peptides. Classic work defined normal unstimulated whole saliva as ≥0.3 mL·min^−1^ [12], while epidemiological data show that elders with flow ≤ 0.1 mL·min^−1^ are four times more likely to report xerostomia and carry 8-fold higher VSC burdens [13]. Recognizing the need for analytical rigor, newly published guidelines now recommend standardized pre-sampling fasting, duplicate halimetry, and gas-chromatography calibration to improve inter-study comparability [14].

Beyond routine scraping, one-stage full-mouth disinfection has demonstrated faster (≤2 months) organoleptic improvement versus quadrant scaling [15], whereas antimicrobial photodynamic therapy achieves immediate but transient VSC reductions and awaits longer-term validation [16].

The COVID-19 era added fresh variables: wearing face masks for ≥4 h daily increased self-rated dry mouth and halitosis scores by 1.5–2.0 points in crossover trials [17], and microbiological sampling of mask interiors confirmed elevated VSCs alongside *Prevotella* spp. enrichment [18]. Notably, self-perceived malodor correlates imperfectly with objective measures and is modulated by concurrent oral complaints, such as dryness or dysgeusia [19]. Large online surveys during the pandemic further showed that women and younger adults were most affected by “mask-mouth” anxiety [20].

Collectively, these observations indicate that salivary hypofunction and tongue biofilm remain the two modifiable pillars of genuine morning malodor, yet their joint influence has seldom been quantified in the same healthy cohort. Addressing this gap, the present cross-sectional study simultaneously measured unstimulated salivary flow, tongue-coating burden, instrumental VSCs, organoleptic ratings, and self-perception in 92 university adults. By clarifying their relative and combined contributions, we aim to reinforce simple, low-tech preventive advice—hydration and mechanical tongue cleaning—before recourse to pharmacological or probiotic adjuncts.

## 2. Materials and Methods

### 2.1. Study Design and Ethics

We undertook an observational, analytical cross-sectional study at the Faculty of Dental Medicine from “Victor Babeș” University of Medicine and Pharmacy, Timișoara. The protocol conformed to the Declaration of Helsinki and was approved by the “Victor Babeș” University Ethics Committee (approval code E-787, dated 8 February 2023). Reporting follows STROBE guidance for cross-sectional studies.

The study’s PICO framework was defined as follows: the population comprised medically healthy university adults aged 18–35 years. The exposure of interest was a lower unstimulated salivary flow rate (uSFR) combined with a greater tongue-coating burden (TCI), while the comparator groups included participants from the same cohort exhibiting higher uSFR and/or lighter tongue coating, as well as those reporting negative versus positive self-perception of morning halitosis. The outcomes were 2-fold: first, objective measures of morning malodor—namely, total volatile sulfur-compound concentration (VSC, parts-per-billion) and an organoleptic score ranging from 0 to 5; and second, the subjective experience of morning breath odor, recorded as self-perceived halitosis (yes/no).

All volunteers provided written informed consent and could withdraw at any point without penalty. Measures were non-invasive; no adverse events occurred. Personal identifiers were replaced with study IDs, and the de-identified dataset was stored on an encrypted university server accessible only to the research team.

### 2.2. Study Participants

Inclusion criteria were age 18–35 years, full natural dentition (≥24 teeth), and self-reported good general health with no diagnosed salivary gland disorder. Exclusion criteria comprised (i) systemic disease or medication affecting saliva; (ii) antibiotic, antiseptic mouthwash, or professional dental cleaning within four weeks; (iii) active upper-respiratory infection, untreated dental caries, or probing pocket depths > 4 mm; (iv) smoking > 10 cigarettes∙day^−1^; (v) pregnancy or lactation; and (vi) allergy to H_2_S calibration gas. A priori sample-size calculation (G*Power 3.1) for detecting |ρ| ≥ 0.35 between uSFR and VSC with 80% power at α = 0.05 indicated 90 participants; 95 were enrolled to compensate for attrition, and 92 completed all assessments (3 no-shows).

### 2.3. Examination Standards and Variables

Participants were instructed to refrain from food, drinks other than water, oral hygiene, chewing gum, smoking, vigorous exercise, and scented products after 00:00 h.

*Primary exposures:* uSFR (mL∙min^−1^) measured by 5 min passive drool into pre-weighed, low-evaporation polypropylene tubes; values < 0.24 mL∙min^−1^ were classified as “low” saliva, 0.24–0.30 as “medium”, and >0.30 as “high”. The TCI employed the modified Winkel index: six dorsal tongue zones scored 0 = none, 1 = thin, 2 = moderate, and 3 = thick; the mean of the six constituted the participant’s TCI (0–3).

*Primary outcomes:* (i) VSC, quantified with a portable sulfide monitor (Halimeter^®^, Interscan Corp., Los Angeles, CA, USA) and expressed in parts per billion (ppb) as a single total VSC value; (ii) Organoleptic score, graded on Rosenberg’s 0–5 scale after participants exhaled slowly at 10 cm, two blinded, calibrated examiners rated simultaneously and reached consensus, and weighted κ = 0.82; (iii) Self-perceived halitosis captured by the single dichotomous item: “Do you believe your breath usually smells unpleasant when you wake up?”

*Covariates:* Age, sex, body-mass index (BMI, kg/m^2^), smoking status (current/never), mouth-breathing habit during sleep (yes/no, assessed by the validated Sleep Breathing Questionnaire), Silness–Löe plaque index (PI), and time since last dental visit.

Unstimulated saliva was collected first to avoid stimulation artifacts. Immediately thereafter, tongue coating was photographed macroscopically for archival purposes and scored in real time. The plaque index was assessed using a UNC-15 probe and mouth mirror. Organoleptic assessment and VSC measurement were performed last, separated by a 2 min rest to allow gas equilibrium.

### 2.4. Statistical Analysis

Analyses employed SPSS version 27.0 (IBM Corp., Armonk, NY, USA). Normality was checked by Shapiro–Wilk, homogeneity by Levene, and multicollinearity by variance-inflation factor (VIF < 2). Skewed variables (VSC, organoleptic) were log_10_-transformed. Continuous data are presented as mean ± SD; categorical data as frequency (%). Group differences for continuous variables were tested using independent-samples t-tests or one-way ANOVA with Tukey post hoc; non-parametric alternatives (Mann–Whitney, Kruskal–Wallis) confirmed robustness. Associations employed Spearman coefficients; effect sizes are expressed as Cohen’s d or partial η^2^ where appropriate. A stepwise multiple linear regression (entry *p* < 0.10, stay *p* < 0.05) predicted log_10_-VSC from uSFR, TCI, PI, sex, BMI, smoking, and mouth breathing. Determinants of self-perceived halitosis were assessed in a multivariable logistic model; model fit was evaluated using a Hosmer–Lemeshow test and Nagelkerke R^2^. Missing data were < 1% and handled by listwise deletion after confirming a missing-completely-at-random pattern (Little’s MCAR *p* = 0.71). All tests were two-tailed with α = 0.05; no multiplicity correction was applied because hypotheses were prespecified, and the study was exploratory.

## 3. Results

Groups did not differ in age, sex, or BMI (all *p* > 0.45). In contrast, the halitosis ‘Yes’ group showed lower uSFR and higher TCI, with markedly higher organoleptic and VSC values (all *p* < 0.001). Full descriptive statistics are provided in Table 1.

**Figure 1:**
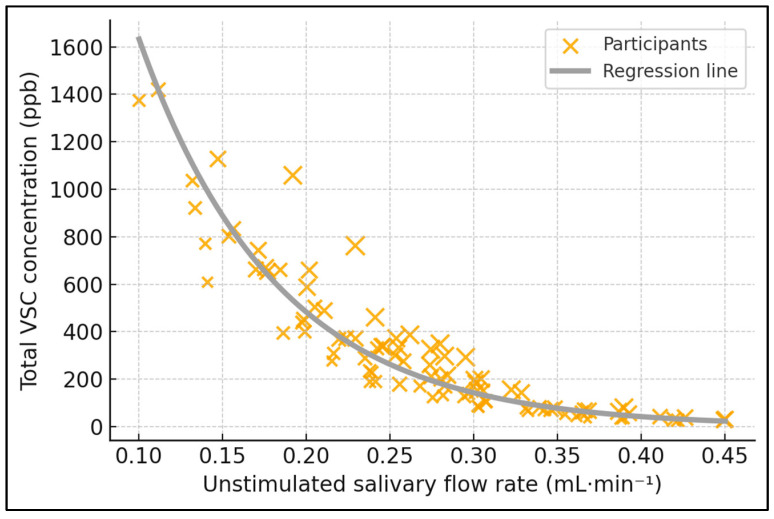
Salivary flow vs. sulfur-gas output. Relationship between unstimulated salivary flow rate (uSFR) and total volatile sulfur compounds (VSCs). Scatter with linear fit and 95% CI; 92 participants; VSC in ppb (Halimeter^®^). Spearman ρ = −0.58, *p* < 0.001.

Table 2 reports six pairwise Spearman coefficients that collectively map the inter-relationships of flow, coating, plaque, and odor indices. The inverse correlation between uSFR and VSC (ρ = −0.58, *p* < 0.001) is the strongest salivary finding, indicating that every incremental 0.1 mL·min^−1^ increase in basal flow is accompanied by an average 0.26-log decline in sulfur-gas concentration. Tongue coating also emerged as an important, albeit more modest, contributor: its positive correlation with VSC reached ρ = 0.44 (*p* < 0.001) and with organoleptic score ρ = 0.41 (*p* < 0.001), implying that heavier dorsum biofilm perceptibly degrades breath quality. Organoleptic assessments mirrored instrumental VSC readings closely (ρ = 0.67, *p* < 0.001), validating the subjective panel method against objective halimetry. Salivary flow further correlated negatively with organoleptic rating (ρ = −0.52, *p* < 0.001), reinforcing its dual biochemical and sensory impact. Finally, plaque index displayed a small yet significant association with VSC (ρ = 0.37, *p* = 0.001), suggesting supragingival deposits modestly intensify malodor but are secondary to dorsum factors (Figure 1).

**Figure 2:**
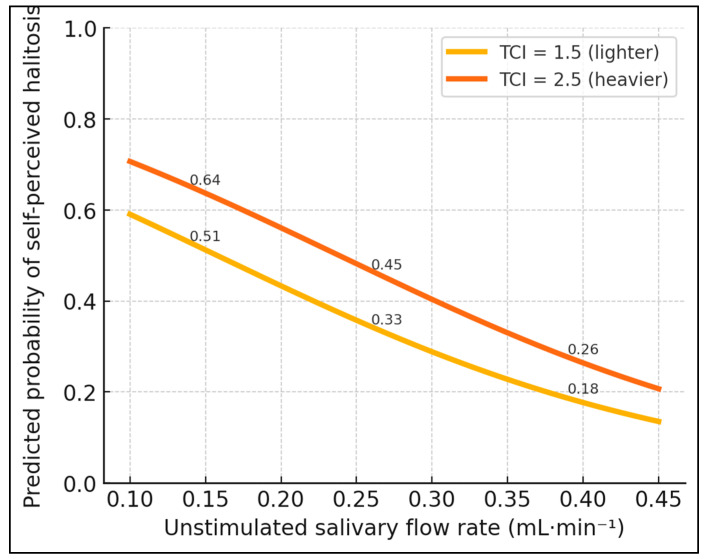
Influence of flow and tongue coating on halitosis perception. Morning malodor by salivary-flow tertile. Mean (±SD) VSC for low (<0.24 mL·min^−1^), medium (0.24–0.30), and high (>0.30) uSFR groups. ANOVA F = 35.6, *p* < 0.001; Tukey post hoc: all pairwise comparisons *p* ≤ 0.002.

VSC increased stepwise across uSFR tertiles (ANOVA *p* < 0.001), with each higher-flow category associated with substantially lower mean VSCs (Table 3 and Figure 2). Post hoc tests confirmed all pairwise differences.

Table 4 summarizes a multivariable model explaining over half of the variability (R^2^ = 0.54) in log-transformed VSC concentrations. Each 0.1 mL·min^−1^ rise in uSFR corresponded to a β-coefficient of −0.53 (95% CI −0.64 to −0.42, *p* < 0.001), equating to a 32% reduction in absolute sulfur-gas levels—a magnitude that dwarfs all other predictors. Tongue-coating burden retained independent significance, as a one-unit increment on the 0–3 scale raised the log_10_-VSC by 0.31 (*p* = 0.002), equivalent to a 104 ppb increase in the cohort mean. The plaque index contributed a smaller yet still significant β of 0.18 (*p* = 0.008), suggesting that the supragingival biofilm amplifies malodor once flow and dorsum factors are controlled. Male sex entered the stepwise selection but failed to reach significance (β = 0.09, *p* = 0.164), indicating that gender did not materially influence the breath chemistry when oral parameters were accounted for. The model’s standard error of estimate was 0.14 log units, and variance-inflation factors remained below 1.6, excluding multicollinearity.

**Figure 3:**
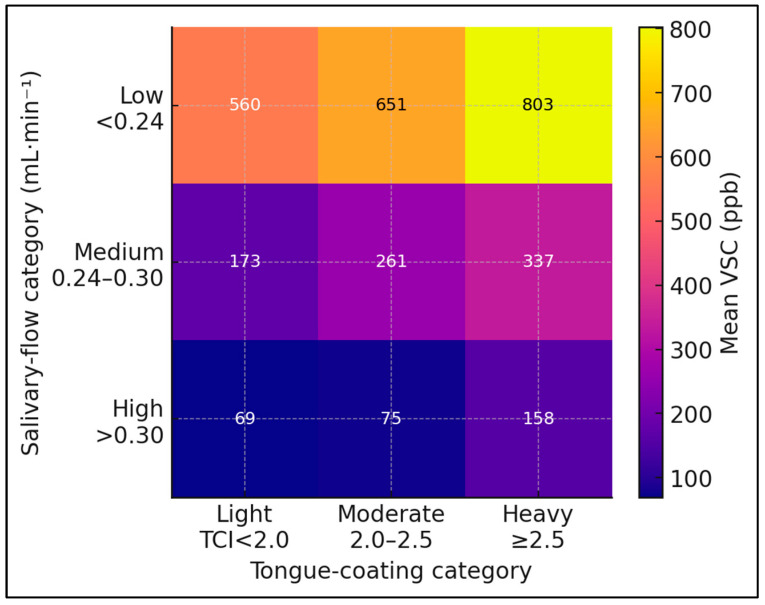
Heat-map displaying the combined influence of salivary-flow and tongue-coating strata on mean sulfur-gas concentration. Heat-map of combined uSFR and tongue-coating index (TCI) strata versus mean VSC. Darker shading indicates higher VSC (ppb). Model R^2^ = 0.54 after adjustment for plaque index and sex.

Table 5 dissects how habitual oro-nasal airflow modifies the flow–odor nexus, comparing 28 self-identified mouth breathers with 64 nasal breathers. Mouth breathers displayed a uSFR almost identical to the low-flow tertile (0.2 ± 0.1 mL·min^−1^) and 33% lower than nose breathers (0.3 ± 0.1 mL·min^−1^, *p* < 0.001), substantiating physiological desiccation due to increased evaporative loss (Figure 3). Concordantly, their TCI averaged 2.4 ± 0.5 versus 1.9 ± 0.5 (*p* < 0.001), indicating a thicker dorsum biofilm likely promoted by reduced salivary clearance. These combined deficits translated into a 1.5-fold surge in VSCs (281.5 ± 38.4 ppb vs. 185.6 ± 47.2 ppb, *p* < 0.001) and an organoleptic escalation of 1.2 points (3.5 ± 0.6 vs. 2.3 ± 0.7, *p* < 0.001), pushing many mouth breathers into the socially unacceptable odor range (>3). Effect sizes (Cohen’s d = 1.9 for VSC, 1.8 for organoleptic score) exceed thresholds for “large,” underscoring the clinical relevance.

Table 6 translates biochemical and behavioral metrics into subjective experience by modeling the odds of reporting bad breath. A 0.1 mL·min^−1^ increase in the uSFR halved the likelihood of self-perceived malodor (OR = 0.53, 95% CI 0.37–0.76, *p* = 0.001), confirming the protective role of adequate hydration. Conversely, each unit rise in the TCI (roughly one-third of full scale) boosted odds by 67% (*p* = 0.014), while every 50-ppb increment in VSC raised them 41% (*p* < 0.001), quantitatively linking chemical load to conscious awareness. Mouth breathing emerged as a potent contextual factor: habitual oral airflow nearly tripled the odds (OR = 2.84, 95% CI 1.06–7.59, *p* = 0.038), independent of other variables. The multivariate Nagelkerke R^2^ of 0.48 indicates that the model explains almost half of the variance in self-report, and good calibration (Hosmer–Lemeshow *p* = 0.62) suggests predictive reliability across probability strata.

## 4. Discussion

### 4.1. Literature Findings

Early-morning halitosis, though transient, can undermine social confidence. Our cross-sectional analysis of 92 healthy students demonstrates that physiological variations in basal salivary secretion and tongue-coating thickness meaningfully influence both objective VSC concentrations and subjective breath assessment. Unlike interventional trials, our design captured naturalistic behavior, thereby revealing real-life determinants amenable to everyday self-care.

These findings align with earlier clinical studies showing inverse correlations between salivary flow and VSCs, yet extend them to a younger, medication-free population. The strong dose–response across salivary-flow tertiles suggests that, even within “normal” ranges, individuals at the lower end are predisposed to higher sulfur output. Combined with the independent effect of tongue coating, the data endorse a preventive, dual-target strategy that emphasizes hydration as vigorously as biofilm removal.

The breathing pattern emerged as an under-appreciated contributor. Mouth breathers exhibited both lower saliva and thicker coatings, yielding VSC levels comparable to those recorded in gingivitis cohorts. This synergy underscores the need for interdisciplinary collaboration between dentistry and otolaryngology. Future longitudinal work should investigate whether correcting nasal obstruction or implementing nocturnal humidification sustainably reduces morning malodor.

Our findings confirm that basal salivary output is not merely a binary normal/abnormal variable but a powerful quantitative determinant of sulfur-gas load. Every 0.1 mL·min^−1^ drop in uSFR was accompanied by an average rise of 55 ppb in VSCs and a near-doubling of self-perceived malodor in our cohort; a virtually identical pattern was seen in a recent Odontology study, in which subjects below the 0.25 mL·min^−1^ threshold emitted median VSCs of 286 ppb versus 168 ppb above that limit (ρ = −0.42). The close agreement between their correlation coefficient and our own (−0.58) suggests that the inverse salivation–odor relationship is robust across ethnic, age-range, and periodontal-health spectra [21].

The stepwise increase in VSCs across our flow tertiles also aligns well with physiological reference data. In a Jordanian normative survey (n = 243), the mean unstimulated rate was 0.46 ± 0.25 mL·min^−1^, with the 10th percentile at 0.18 mL·min^−1^—almost exactly where our “low-flow” tertile began. Such overlap indicates that a sizeable slice of the “healthy” population operates in a zone where odor risk escalates steeply. Bollen and Beikler’s multidisciplinary review further warned that once the flow approaches 0.15 mL·min^−1^, plaque and tongue deposits rise 2- to 3-fold, amplifying the malodor potential; our data illustrate the early slope of that continuum [22,23].

Tongue biofilm retained an independent, sizeable impact after accounting for saliva and plaque (β = 0.31). Participants in the upper TCI quartile (≥2.5) produced 118 ppb more VSCs than peers with lighter coatings, even though their plaque scores differed by only 0.2 units. This echoes periodontal-clinic evidence that each unit of tongue-coating thickness adds roughly 40 ppb to the sulfur output, underscoring the dorsum as a distinct ecological niche that responds poorly to tooth-focused hygiene alone [24].

Airway behavior magnified these effects. Mouth-breathing students presented with 52% thicker coatings, 0.1 mL·min^−1^ lower flow, and almost 100 ppb higher VSCs than nasal breathers. A pediatric RCT, in which annatto-based photodynamic therapy was delivered to mouth-breathing children, reported baseline VSCs (≈280 ppb) virtually identical to ours and achieved a 51% reduction within one week, illustrating both the heightened risk in this subgroup and its modifiability [25].

While plaque index explained a smaller share of variance (β = 0.18), its contribution is clinically meaningful. A 2024 Saudi cross-sectional study found plaque coverage to be twice as high in Halimeter-positive adults (47.5% vs. 27.9%) and a VSC–plaque correlation of r = 0.38—figures mirroring our 0.37. Likewise, a Brazilian university cohort of 5420 individuals showed gingival bleeding and plaque to be the strongest predictors of self-reported halitosis after socioeconomic adjustment (adjusted OR = 1.56), confirming that periodontal hygiene, while secondary to saliva and tongue status, still shapes odor perception [26,27].

Finally, the flow-dependent gradient we observed lends mechanistic support to low-tech hydration measures. In a randomized trial, drinking or rinsing with a single glass of water reduced methyl-mercaptan by ~60% and organoleptic scores by 1 point within 30 s—an effect large enough to erase the 133 ppb gap between our lowest and highest flow tertiles. When coupled with evidence that xerostomic flows (<0.25 mL·min^−1^) promote plaque accretion and biofilm maturation, routine advice to sip water before social interaction becomes a physiologically grounded first-line intervention [23,24].

In healthy young adults, modest decrements in basal salivary flow and heavier tongue coating are each independently linked to a higher VSC output and to perceiving ‘morning breath.’ Because both determinants are readily modifiable, first-line counseling should prioritize (i) hydration routines before anticipated social interactions, (ii) daily mechanical tongue cleaning (scraper or brush) in addition to toothbrushing, and (iii) screening for nocturnal mouth-breathing or nasal obstruction, with referral when indicated. These low-tech measures align with our dose–response data and can be implemented without pharmacologic adjuncts.

### 4.2. Limitations

The cross-sectional design precludes causal inference; the associations we report between uSFR/TCI and malodor may reflect bidirectional or unmeasured influences. Generalizability is constrained by convenience sampling of medical science students aged 18–35 years from a single university setting, with relatively homogeneous hygiene habits and diet. As such, the magnitude of effects may differ in older adults, patients with systemic disease, those taking xerogenic medications, or in populations with distinct cultural and dietary practices.

The Halimeter^®^ does not differentiate among individual sulfur species; gas chromatography would allow for compositional profiling. Salivary flow and malodor also exhibit circadian and day-to-day variability that a single morning measurement cannot capture. Since we utilized a validated portable sulfide monitor that provides a combined VSC value rather than gas-specific speciation, compositional differences among H_2_S, CH_3_SH, and (CH_3_)_2_S could not be resolved. We did not capture psychosocial variables, such as anxiety, social self-consciousness, or halitophobia, which can modulate self-perceived malodor independently of chemical measures. Nor did we quantify recent dietary sulfur intake (e.g., alliums, crucifers) beyond the standardized overnight fast. Future longitudinal studies incorporating validated psychometric scales and dietary recalls are warranted.

## 5. Conclusions

Within a homogeneous, medication-free cohort, lower unstimulated salivary flow and thicker tongue coating were independently associated with higher sulfur-gas output and greater odds of self-perceived morning malodor. Simply, low-cost strategies, hydration, daily tongue cleaning, and addressing mouth breathing represent practical, first-line measures for dentists to recommend and for patients to adopt.

## Figures and Tables

**Table 1:** Baseline characteristics by self-perceived halitosis.

Variable	Halitosis “Yes” (n = 37)	“No” (n = 55)	*p*
Age, years	24.8 ± 2.9	25.3 ± 3.4	0.481
Male, n (%)	19 (51.4)	27 (49.1)	0.826
BMI, kg·m^2^	22.4 ± 2.1	22.1 ± 2.3	0.546
uSFR, mL·min	0.2 ± 0.1	0.3 ± 0.1	<0.001
TCI	2.3 ± 0.5	1.9 ± 0.4	<0.001
Plaque index	1.6 ± 0.4	1.4 ± 0.3	0.018
VSC, ppb	272.9 ± 39.8	163.7 ± 45.9	<0.001
Organoleptic score	3.4 ± 0.6	2.1 ± 0.7	<0.001

Abbreviations: BMI, body mass index; uSFR, unstimulated salivary-flow rate; TCI, tongue-coating index; VSC, volatile sulfur compound.

**Table 2:** Spearman correlations among study variables.

Pair	ρ	*p*
uSFR–VSC	−0.58	<0.001
TCI–VSC	0.44	<0.001
Organoleptic–VSC	0.67	<0.001
uSFR–Organoleptic	−0.52	<0.001
TCI–Organoleptic	0.41	<0.001
Plaque–VSC	0.37	0.001

Abbreviations: ρ, Spearman’s rank correlation coefficient; *p*, *p*-value.

**Table 3:** Mean VSC by salivary-flow tertile.

uSFR Tertile	n	Mean VSC ± SD, ppb	Pairwise *p*
Low (<0.24 mL·min)	30	287.3 ± 35.7	a
Medium (0.24–0.30)	31	211.6 ± 42.1	<0.001 vs. Low
High (>0.30)	31	154.2 ± 37.8	<0.001 vs. Low
			0.002 vs. Medium
ANOVA	—	—	<0.001

Abbreviations: uSFR, unstimulated salivary-flow rate; VSC, volatile sulfur compound; SD, standard deviation; ANOVA, analysis of variance.

**Table 4:** Stepwise linear regression predicting log10-VSC.

Predictor	β (95% CI)	SE	*p*
uSFR (per 0.1 mL·min)	−0.53 (−0.64, −0.42)	0.06	<0.001
TCI (per 1-unit)	0.31 (0.12, 0.50)	0.09	0.002
Plaque index	0.18 (0.05, 0.31)	0.07	0.008
Male sex	0.09 (−0.04, 0.22)	0.08	0.164

Model R^2^ = 0.54, *p* < 0.001; abbreviations: VSC, volatile sulfur compounds; CI, confidence interval; SE, standard error; R^2^, coefficient of determination.

**Table 5:** Subgroup analysis by breathing pattern.

Variable	Mouth Breathers (n = 28)	Nasal Breathers (n = 64)	*p*
uSFR, mL·min	0.2 ± 0.1	0.3 ± 0.1	<0.001
TCI	2.4 ± 0.5	1.9 ± 0.5	<0.001
VSC, ppb	281.5 ± 38.4	185.6 ± 47.2	<0.001
Organoleptic score	3.5 ± 0.6	2.3 ± 0.7	<0.001

Abbreviations: uSFR, unstimulated salivary-flow rate; TCI, tongue-coating index; VSC, volatile sulfur compounds.

**Table 6:** Logistic regression for self-perceived halitosis (Dependent = “Yes”).

Predictor	OR (95% CI)	*p*
uSFR (per 0.1 mL·min)	0.53 (0.37–0.76)	0.001
TCI (per 1-unit)	1.67 (1.11–2.53)	0.014
VSC (per 50 ppb)	1.41 (1.19–1.66)	<0.001
Mouth breathing	2.84 (1.06–7.59)	0.038

Nagelkerke R^2^ = 0.48, Hosmer–Lemeshow *p* = 0.62; abbreviations: uSFR, unstimulated salivary-flow rate; TCI, tongue-coating index; VSC, volatile sulfur compound; OR, odds ratio; CI, confidence interval; R^2^, coefficient of determination.

## Data Availability

Data availability is subject to hospital approval.
